# Peer Review of “Effect of Thermal and Vibration Changes on Automated External Defibrillator Circuit Boards: Finite Element Analysis Study”

**DOI:** 10.2196/80137

**Published:** 2025-08-19

**Authors:** Anonymous

**Keywords:** circuit board, automated external defibrillator, heart, cardiology, vibration, thermal changes, medical devices

*This is a peer-review report for “Effect of Thermal and Vibration Changes on Automated External Defibrillator Circuit Boards: Finite Element Analysis Study.”*

## Round 1 Review

### General Comments

I have read the submitted manuscript [1] to your journal entitled “Analysis of Vibration and Thermal of a Modeled Circuit Board of Automated External Defibrillator (AED) Medical Device.” The author utilized finite element analysis to simulate static and dynamic testings in determining the vibration and thermal effects on the operations of the automated external defibrillator medical device.

Based on the outcomes of this conducted study and the potential benefits from this study, I would recommend this manuscript to be published in your reputable journal after the minor comments are properly addressed.

### Specific Comments

#### Minor Comments

There are 4-member and 8-member supports. The author should provide more evidence on how these affect the analysis results.

This manuscript will benefit from other works on the failure modes of materials. The author is encouraged to expand the Literature Review section and also provide more references.

The grammar should be refined. Some of the grammar in this manuscript should be corrected. For example, “This study was performed to analysis the vibration...” “Analyse.” Fig 14 = Fig. 14 or Figure 14. The unit of temperature is degree c; the *c* is capitalized.

When citing a research paper within the manuscript, it is the first/lead author who should be named in the “Name et al” format; the author should use a consistent citation format.

The author should remove parentheses from the manuscript title: “Analysis of Vibration and Thermal of a Modelled Circuit Board of Automated External Defibrillator Medical Device”

For reproducibility by other researchers, the author should consider providing simulation data as supplementary information.

The author should properly cite other works where applicable. For example, “From other research results, it can be verified that the natural frequencies...” The author needs to insert appropriate citations for comments like these.
